# ERK/Drp1‐dependent mitochondrial fission contributes to HMGB1‐induced autophagy in pulmonary arterial hypertension

**DOI:** 10.1111/cpr.13048

**Published:** 2021-05-04

**Authors:** Wei Feng, Jian Wang, Xin Yan, Qianqian Zhang, Limin Chai, Qingting Wang, Wenhua Shi, Yuqian Chen, Jin Liu, Zhan Qu, Shaojun Li, Xinming Xie, Manxiang Li

**Affiliations:** ^1^ Department of Respiratory and Critical Care Medicine the First Affiliated Hospital of Xi’an Jiaotong University Xi’an, Shaanxi China

**Keywords:** autophagy, bone morphogenetic protein receptor 2, dynamin‐related protein 1, extracellular signal‐regulated kinases 1/2, high mobility group box 1, pulmonary vascular remodelling

## Abstract

**Objectives:**

High‐mobility group box‐1 (HMGB1) and aberrant mitochondrial fission mediated by excessive activation of GTPase dynamin‐related protein 1 (Drp1) have been found to be elevated in patients with pulmonary arterial hypertension (PAH) and critically implicated in PAH pathogenesis. However, it remains unknown whether Drp1‐mediated mitochondrial fission and which downstream targets of mitochondrial fission mediate HMGB1‐induced pulmonary arterial smooth muscle cells (PASMCs) proliferation and migration leading to vascular remodelling in PAH. This study aims to address these issues.

**Methods:**

Primary cultured PASMCs were obtained from male Sprague‐Dawley (SD) rats. We detected RNA levels by qRT‐PCR, protein levels by Western blotting, cell proliferation by Cell Counting Kit‐8 (CCK‐8) and EdU incorporation assays, migration by wound healing and transwell assays. SD rats were injected with monocrotaline (MCT) to establish PAH. Hemodynamic parameters were measured by closed‐chest right heart catheterization.

**Results:**

HMGB1 increased Drp1 phosphorylation and Drp1‐dependent mitochondrial fragmentation through extracellular signal‐regulated kinases 1/2 (ERK1/2) signalling activation, and subsequently triggered autophagy activation, which further led to bone morphogenetic protein receptor 2 (BMPR2) lysosomal degradation and inhibitor of DNA binding 1 (Id1) downregulation, and eventually promoted PASMCs proliferation/migration. Inhibition of ERK1/2 cascade, knockdown of Drp1 or suppression of autophagy restored HMGB1‐induced reductions of BMPR2 and Id1, and diminished HMGB1‐induced PASMCs proliferation/migration. In addition, pharmacological inhibition of HMGB1 by glycyrrhizin, suppression of mitochondrial fission by Mdivi‐1 or blockage of autophagy by chloroquine prevented PAH development in MCT‐induced rats PAH model.

**Conclusions:**

HMGB1 promotes PASMCs proliferation/migration and pulmonary vascular remodelling by activating ERK1/2/Drp1/Autophagy/BMPR2/Id1 axis, suggesting that this cascade might be a potential novel target for management of PAH.

## Graphical abstract

The molecular mechanisms underlying HMGB1‐induced pulmonary vascular remodelling in PAH. HMGB1 increases Drp1 phosphorylation and Drp1‐dependent mitochondrial fission through activation of ERK1/2 signaling pathway, and subsequently stimulates autophagy activation, which further lead to BMPR2 lysosomal degradation and Id1 downregulation, and ultimately promotes PASMCs proliferation/migration and pulmonary vascular remodeling in PAH.

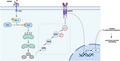

## 1 INTRODUCTION

Pulmonary arterial hypertension (PAH) is a chronic and devastating cardiopulmonary disorder typified by extensive pulmonary vascular occlusion, mainly due to persistent vasoconstriction, excessive vascular remodelling, and thrombosis in situ, leading to progressive pulmonary vascular resistance and eventually right ventricular failure.^1^ Pathologically, pulmonary vascular remodelling is the key structural alteration of PAH.^1^, ^2^ Excessive pulmonary artery smooth muscle cells (PASMCs) proliferation and migration is the prominent feature in vascular remodelling. Owing to current PAH therapies mainly focus on vasodilation, not specially target vascular remodelling, there is an urgent need to better decipher the molecular mechanisms underlying exacerbated PASMCs proliferation and migration processes.

As a critical damage‐associated molecular pattern (DAMP), high‐mobility group box‐1 (HMGB1) is released from apoptotic or necrotic cells and from cells activated by cytokines stimulations.^3^ Once secreted, extracellular HMGB1 promotes proliferation, migration and differentiation by binding to several receptors. HMGB1 has been identified as a biomarker of PAH pathogenesis, with the evidence that HMGB1 levels are highly increased in lungs and serums of PAH patients and positively correlate with disease severity.^4^, ^5^, ^6^ At the same time, circulating HMGB1 levels are elevated in serums of monocrotaline (MCT) or hypoxia induced PAH rodent models, and these elevations are associated with enhanced pulmonary vascular remodelling.^7^, ^8^ Pharmacological inhibition of HMGB1 alleviates pulmonary vascular remodelling in both MCT‐ or Sugen/hypoxia‐induced PAH rat models.^6^, ^8^, ^9^ In addition, HMGB1 promotes proliferation, hypertrophy and migration of PASMCs in vitro.^8^, ^9^, ^10^, ^11^ Collectively, these studies suggest that HMGB1 plays a crucial role in the pathophysiology of PAH. However, the molecular mechanisms underlying how HMGB1 drives PAH pathogenesis remain to be clarified.

Aberrant mitochondrial fragmentation has been reported in PAH development, and this pathological fission is mainly mediated by excessive activation and upregulation of GTPase dynamin‐related protein 1 (Drp1), which further promotes PASMCs proliferation in human and experimental PAH.^12^, ^13^, ^14^ When activated, Drp1 translocates from cytosol to mitochondria, and interacts with binding partners, thereby facilitating mitochondrial division.^15^, ^16^ In particular, Drp1 serine 616 phosphorylation by extracellular signal‐regulated kinase (ERK) 1/2 has been shown to trigger abnormal mitochondrial fission and promote cell proliferation and chemoresistance in several types of cancers.^17^, ^18^ However, to date, whether Drp1 activation and Drp1‐dependent mitochondrial fission are involved in HMGB1‐induced PASMCs proliferation/migration and the mechanisms responsible for the altered mitochondrial dynamics contributing to PASMCs proliferation and migration remain poorly characterized.

Recent studies have demonstrated that PAH development is associated with increased lung autophagy and impaired bone morphogenetic protein receptor 2 (BMPR2) and inhibitor of DNA binding 1 (Id1) expressions.^19^, ^20^, ^21^ It has been reported that HMGB1 released from Resistin‐stimulated pulmonary artery endothelial cells (PAECs) induces BMPR2 reduction in PASMCs.^22^ Further studies have revealed that HMGB1 triggers autophagy for chemoresistance through ERK1/2 signalling pathway activation in colorectal cancer and lung adenocarcinoma.^17^, ^23^ Taken together, these findings lead to our hypothesis that extracellular HMGB1 acts as a pivotal mediator for PASMCs proliferation/migration and pulmonary vascular remodelling, these effects could be mediated by increased Drp1 phosphorylation and Drp1‐dependent mitochondrial fission through ERK1/2 signalling pathway, and subsequently promotes autophagy activation and BMPR2 lysosomal degradation and Id1 downregulation, thereby contributes to PAH development.

## 2 MATERIALS AND METHODS

### 2.1 Cell culture and reagents

PASMCs were isolated from pulmonary arteries of male Sprague‐Dawley (SD) rats (120‐160 g) as previously described.^24^ Cells were cultured with high glucose Dulbecco's modified Eagle medium (DMEM) (Gibco, NY, USA) containing 10% foetal bovine serum (FBS) (Gemini Bio, Woodland, CA, USA) plus 100 U/mL penicillin‐streptomycin. Cells were maintained at 37% in 5% CO_2_ and 95% relative humidity incubator. PASMCs were used in all experiments within six passages. The identification of rat PASMCs was determined by immunostaining for α‐smooth muscle actin (α‐SMA, 1:200) (BM0002, Boster, CA, USA) and immunofluorescence staining confirmed that the cultured cells contained over 95% PASMCs. Before each experiment, cells were made quiescent in serum‐free medium for 24 h. HMGB1 (0‐300 ng/mL) (1690‐HMB050, R&D systems, Minneapolis, USA) was used to stimulate PASMCs. U0126 (10 μM) (MedChemExpress, NJ, USA) was applied to inhibit ERK1/2, and Chloroquine phosphate (CQ, 20 μM) (Aladdin, Shanghai, China) was employed to block autophagy. The concentrations of the compounds were chosen based on previous studies.^8^, ^9^, ^10^, ^11^, ^17^, ^25^, ^26^

### 2.2 Cell proliferation assessment

PASMCs were seeded in 96‐well plates at 2 × 10^3^ cells/well and cultured for 24 h. After serum‐starved for 24 h, cells were incubated with HMGB1 for 24 h. The proliferation of PASMCs was detected by the Cell Counting Kit‐8 (CCK‐8) and EdU incorporation assays. In CCK‐8 experiment, after 24 h incubation with HMGB1, CCK‐8 reagent (1:10) (KGA317, KeyGen Biotech, Nanjing, China) was added into each well and treated for 3 h, and the absorbance was recorded at 450 nm by using a microplate reader (Bio‐Rad, CA, USA). The DNA incorporation rate was tested using the BeyoClick™ EdU‐488Kit (Beyotime, Shanghai, China) according to the instructions and imaged by inverted fluorescence microscope. The number of EdU‐positive cells/total cells were counted with Image J software (NIH, Bethesda, MD, USA).

### 2.3 Cell migration measurements

Migration of PASMCs were determined with wound healing assay and transwell chambers (24‐well, 8‐μm pore size, Corning). In wound healing assay, PASMCs were plated in 6‐well plates to 90% confluence. After an overnight starvation in serum‐free condition, wounds were created with a 200 µl sterile pipette tip. Then, PASMCs were exposed to HMGB1 and images were obtained at 0 h and 24 h post‐wounding. The areas were randomly selected, and widths of wounded areas were measured. In Transwell assay, PASMCs were seeded in the upper chambers with serum‐free medium, and the lower chambers were filled with complete medium containing HMGB1 or not. After incubation for 24 h, the cells in the upper chambers were removed, and the cells that migrated were stained with 0.3% crystal violet, followed by counting under an inverted microscope.

### 2.4 RNA isolation, cDNA synthesis and qRT‐PCR

Total RNA was extracted from cultured cells or lung tissues by using TRIzol (Invitrogen, CA, USA). Isolated RNA was subsequently reverse‐transcribed into cDNA by using PrimeScript™ RT Master Mix (RR036A, TaKaRa, Tokyo, Japan). The generated cDNA was amplified with primer pairs for the indicated gene, to perform qRT‐PCR on Applied Biosystems StepOnePlus Real‐Time PCR System (ThermoFisher Scientific, MA, USA) by using TB Green® Premix Ex Taq™ II (RR820A, TaKaRa, Tokyo, Japan). Quantifications of target genes were normalized to relative levels of β‐actin (#B661202, Sangon Biotech, Shanghai, China) and expressed as ΔCt. The data was analysed using the 2^−ΔΔCt^ method. The primers (Sangon Biotech, Shanghai, China) sequences in this study for rats mRNAs were listed as below: Drp1: F: 5’‐GAGAACTACCTTCCGCTGTATCGC‐3’, R:5’‐CACCATCTCCAATTCCACCACCTG‐3’; BMPR2: F:5’‐CAAAGCCCAGAAGAGCACAGAGG‐3’, R: 5’‐TTGCCATCCTGCGTTGACTCAC‐3’; Id1: F: 5’‐GGCGAAGTGGTGCTTGGTCTG‐3’, R: 5’‐GTAGCAGCCGTTCATGTCGTAGAG‐3’.

### 2.5 siRNA transfection

PASMCs were grown to 30%–50% confluence and then transfected with 25 picomole of siRNA using Lipofectamine^TM^ 2000 regent (Invitrogen, CA, USA). After 6 h transfection, cells were cultured in serum‐containing medium for a period of 24 h for mRNA knockdown or 48 h for protein knockdown. The sequences of siRNA duplexes specific for rat Drp1, BMPR2 and negative control were: Drp1 siRNA, sense 5’‐GGUGCUAGGAUUUGUUAUATT‐3’, anti‐sense 5’‐UAUAACAAAUCCUAGCACCTT‐3’; BMPR2 siRNA, sense 5’‐GGACAAUAUUAUGCUCCAATT‐3’, anti‐sense 5’‐UUGGAGCAUAAUAUUGUCCTT‐3’; negative control (NC) siRNA, sense 5’‐UUCUCCGAACGUGUCACGUTT‐3′, anti‐sense 5’ ‐ACGUGACACGUUCGGAGAAT‐3’; All siRNA was synthesized by GenePharma (Shanghai, China).

### 2.6 Western blotting analysis

As we previously described,^27^ proteins were isolated by using RIPA lysis buffer and separated by 10% and 15% SDS‐PAGE. After being transferred to PVDF membranes, membranes were probed with the following antibodies against: p‐ERK1/2 (1:2000, #4370, Cell Signaling Technology, MA, USA), t‐ERK1/2 (1:1000, #4695, Cell Signaling Technology, MA, USA), p‐Drp1^Ser616^ (1:500, #12749, Signalway Antibody, MA, USA), t‐Drp1(#5391, 1:1000, Cell Signaling Technology, MA, USA), Beclin1 (1:500, sc‐48381, Santa Cruz Biotechnology, TX, USA), p62 (1:1000, 18420‐1‐AP, Proteintech Group, Wuhan, China), LC3B (1:300, 18725‐1‐AP, Proteintech Group, Wuhan, China), BMPR2 (1:500, BS7659, Bioworld Technology, MN, USA), Id1(1:500, BS0047, Bioworld Technology, MN, USA) and β‐actin (1:1000, YM3028, Immunoway, TX, USA) at 4℃ overnight, and then re‐blotted with horseradish peroxidase‐labelled secondary antibodies (anti‐mouse, ZhuangzhiBio, EK010, 1:5000; anti‐rabbit, ZhuangzhiBio, EK020, 1:5000) at room temperature for 1 h. Bioluminescence was detected with Image Lab software (Bio‐rad, CA, USA) and quantified by Image J software.

### 2.7 MitoTracker Red fluorescence staining

Cells were stained with 200 nM MitoTracker® Red CMXRos (MTR) (M9940, Solarbio, Beijing, China) in completed medium for 30 min at 37℃. The samples were then imaged under a confocal laser scanning fluorescence microscope. The excitation/emission wavelengths were 579/599 nm.

### 2.8 Transmission electron microscopy

As described previously,^28^ PASMCs and lung tissues from rats were fixed by glutaraldehyde, postfixed with OsO_4_, dehydrated by alcohol and then embedded in araldite. Seventy nanometer sections were sliced from the specimens, and stained with uranyl acetate and lead citrate. The ultrastructure evaluations were performed using a transmission electron microscope (TEM) (H‐7650, Hitachi, Japan).

### 2.9 Animal experiments

All the procedures were performed on approval by the Institutional Animal Ethics Committee of Xi'an Jiaotong University and following the Guide for the Care and Use of Laboratory Animals of Xi'an Jiaotong University Animal Experiment Center. Male SD rats were purchased from Xi'an Jiaotong University Experimental Animal Center. All rats were kept on a 12 h light/dark cycle environment and fed ad libitum with a standard diet at temperature of 20 ± 2℃. Briefly, rats (weighing approximately 200‐220 g) were randomly divided into five groups (n = 8 animals/group) and treated as below: CON group: received saline by intraperitoneal (ip) injection on day 1, and then with an equal volume of vehicle (0.9% NaCl) alone for 28 days; MCT group: received a single ip injection of 60 mg/kg MCT (Must Bio‐Technology, Chengdu, China) at day 1 to induce PAH, as previously described^27^ ; MCT+DMSO group: received vehicle DMSO by daily ip injection; MCT+Glycyrrhizin (GLY) group: received GLY (100 mg/kg, 53956‐04‐0, Santa Cruz, CA, USA) by daily ip injection^8^, ^9^ ; MCT+Mitochondrial division inhibitor (Mdivi‐1) group: received Mdivi‐1 (50 mg/kg, HY‐15886, MedChemExpress, NJ, USA) by twice weekly ip injection ^12^ ; MCT+CQ group: received CQ (50 mg/kg, Aladdin, Shanghai, China) by daily gavage tube.^19^

### 2.10 Hemodynamic and RV hypertrophy measurements

After four weeks interventions, rats were euthanized for hemodynamic measurements as described previously.^24^ All rats underwent closed‐chest right heart catheterization to assess right ventricle systolic pressure (RVSP) and mean pulmonary artery pressure (mPAP). For RV hypertrophy, the RV and left ventricle (LV) plus interventricular septum (S) were dissected and measured to detect the RV/LV+S ratio (ie Fulton index).

### 2.11 Histological and immunohistochemistry staining

After haemodynamic study and exsanguination, lung tissues from marginal right upper lobes were fixed in 4% paraformaldehyde at room temperature overnight, and then embedded in paraffin wax. These lung tissues were prepared as 5 μm thick sections and detected with haematoxylin–eosin (HE) staining or Elastin van Gieson (EVG) staining as previously reported.^9^, ^27^, ^29^, ^30^ For pulmonary arterioles (PAs) vascular remodelling, the media wall thickness of vessels (20–70 μm diameters, n = 10 per rat) was assessed by a light microscope (cellSens Imaging Software, Olympus, Tokyo, Japan) as reported.^27^, ^31^ Immunohistochemistry staining for α‐SMA (1:200, BM0002, Boster, CA, USA) and Ki67 (1:200, YM3064, Immunoway, TX, USA) were also performed to detect PAs muscularization and PASMCs proliferation, respectively, as previously described.^19^, ^32^, ^33^

### 2.12 Statistical analysis

Data were presented as mean ± standard error of mean (SEM). All data passed normality and equal variance tests with the Shapiro–Wilk test and F test, respectively. Student's *t*‐test was conducted for comparisons between two groups, and one‐way ANOVA followed by Newman–Keuls *post‐hoc* test was performed for multiple comparisons. All statistical analyses were processed using Prism version 8.0 (GraphPad Software, La Jolla, CA, USA). *P* value <.05 was determined statistically significant.

## 3 RESULTS

### 3.1 HMGB1 increases Drp1 phosphorylation, mitochondrial fragmentation, proliferation and migration in PASMCs

**FIGURE 1:**
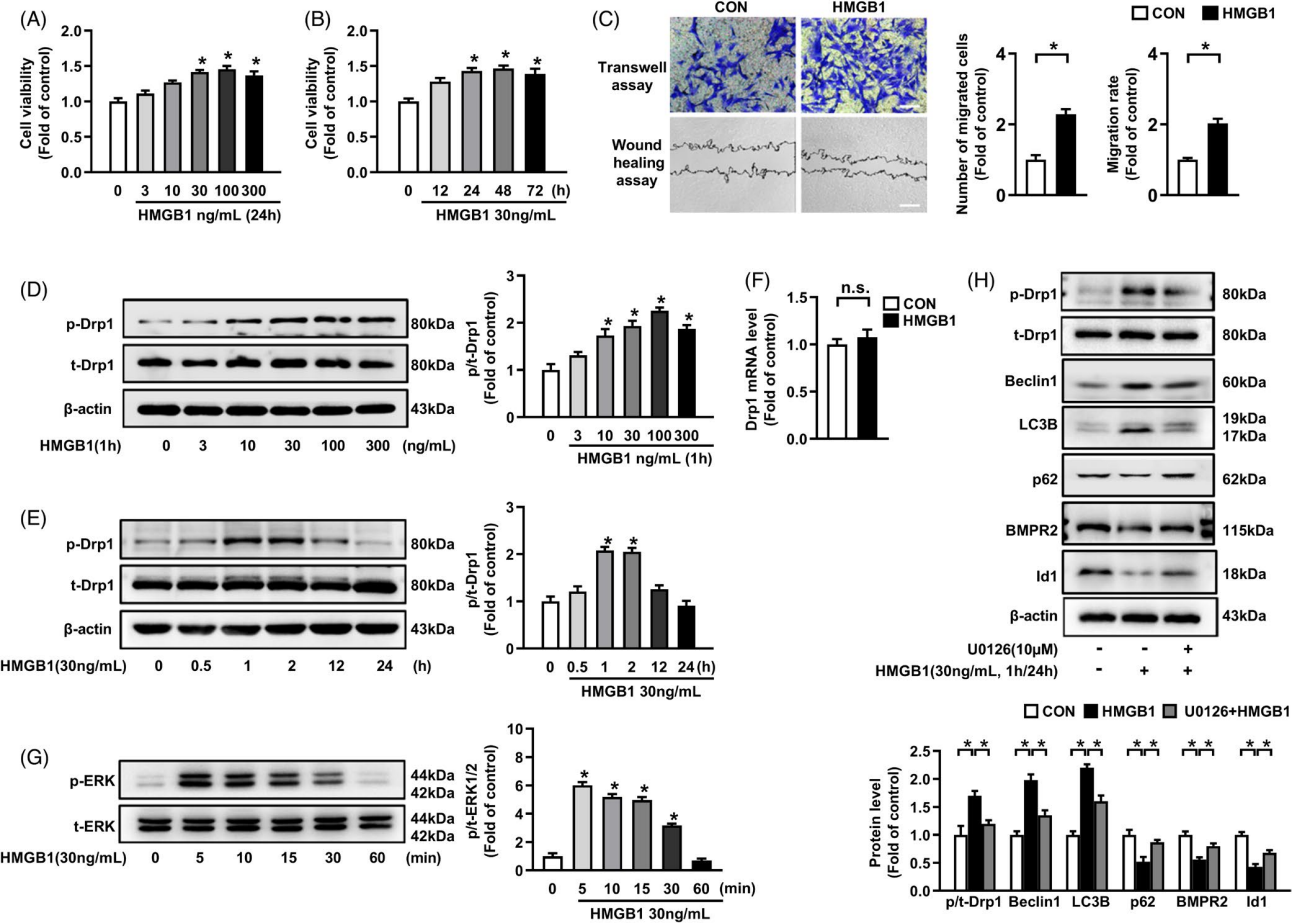
HMGB1 stimulates PASMCs proliferation and migration, and promotes Drp1 phosphorylation, autophagy activation, BMPR2/Id1 downregulation by ERK1/2 activation. A, PASMCs were exposed in different concentrations (0‐300 ng/mL) of HMGB1 for 24 h, cell viability was measured by Cell Counting Kit‐8 (CCK‐8) assay. B, PASMCs were exposed to 30 ng/mL HMGB1 for the indicated time (0‐72 h), and cell viability was analysed using CCK‐8 assay. C, PASMCs were incubated with 30 ng/mL HMGB1 for 24 h, and cell migration was detected using Transwell assay (upper panel, scale bar =200 μm) and Wound healing assay (lower panel, scale bar = 400 μm). D, p‐Drp1 and t‐Drp1 levels were evaluated by immunoblotting in PASMCs with different concentrations (0‐300 ng/mL) of HMGB1 for 1 h. E, p‐Drp1 and t‐Drp1 levels were examined by immunoblotting in PASMCs with 30 ng/mL HMGB1 for indicated time (0‐24 h). F, PASMCs were intervened with 30 ng/mL HMGB1 for 24 h, and Drp1 mRNA level was examined by qRT‐PCR. G, HMGB1 (30 ng/mL) time‐dependently activated ERK1/2 signalling pathway in PASMCs. H, PASMCs were pre‐treated with 10 μM U0126 for 30 min and then stimulated by 30 ng/mL HMGB1 for 1 h, protein levels of p‐Drp1 and t‐Drp1 were detected by immunoblotting. Cells were pre‐treated with 10 μM U0126 for 30 min and then incubated with 30 ng/mL HMGB1 for 24 h, protein levels of Beclin1, LC3B, p62, BMPR2 and Id1 were assessed by immunoblotting. *P < .05

Firstly, we investigated the effect of HMGB1 on induction of PASMCs proliferation and migration. As depicted in Figure 1A, HMGB1 (30‐300 ng/mL) dose‐ dependently stimulated PASMCs proliferation at 24 h compared to control group, whereas no statistical significance was observed in cells treated with low concentrations of HMGB1 (3‐10 ng/mL). Based on the result, 30 ng/mL HMGB1 was used in subsequent cell experiments. Figure 1B showed that HMGB1 promoted PASMCs proliferation in a time‐dependent manner. In addition, we further examined the effect of HMGB1 (30 ng/mL, 24 h) on PASMCs migration by Transwell and wound healing assays. The results demonstrated the numbers of migrated cells in HMGB1 group were significantly more than that in control group, suggesting that HMGB1 significantly increases PASMCs migration (Figure 1C).

**FIGURE 2:**
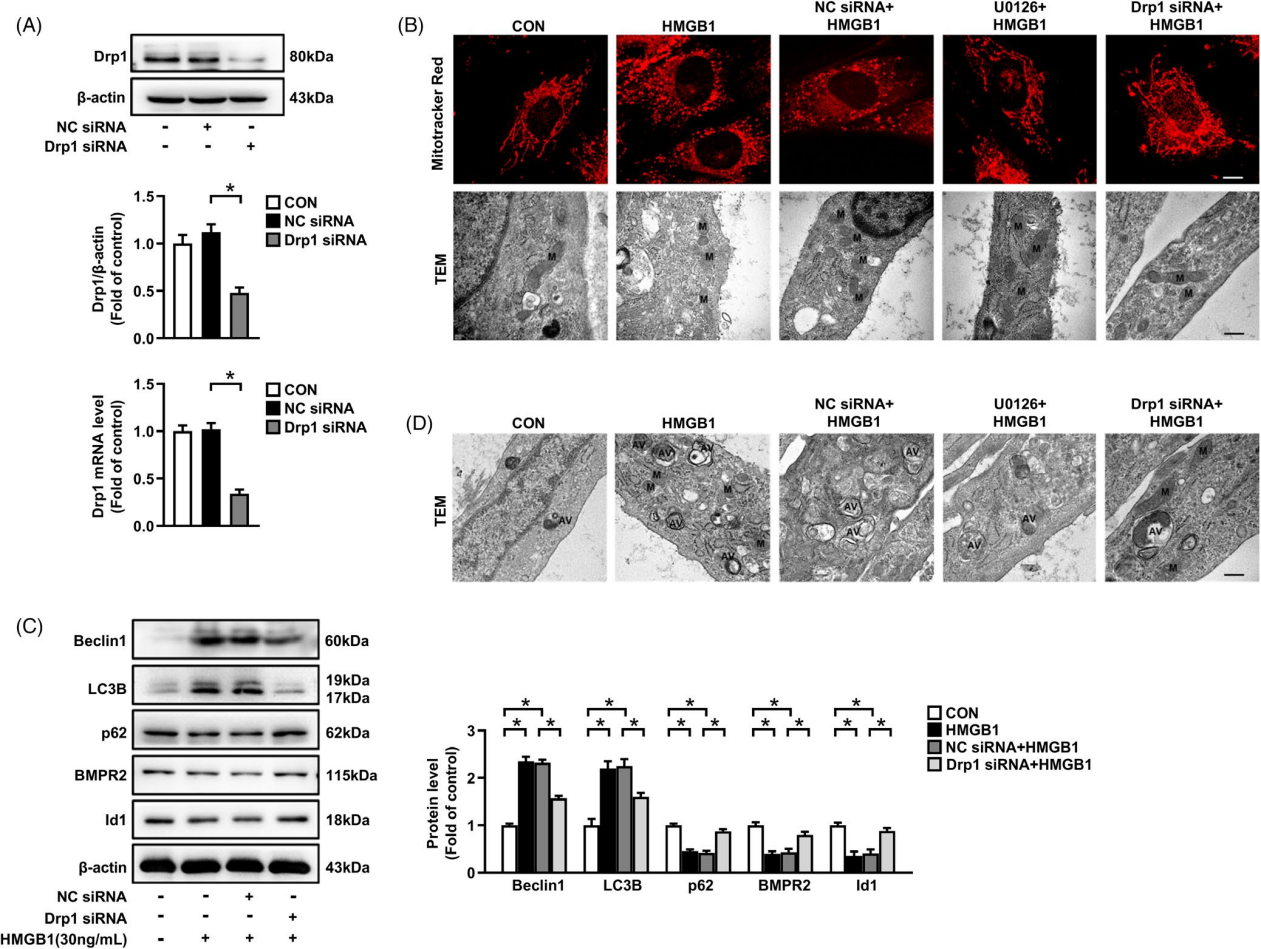
Drp1 knockdown prevents HMGB1‐induced mitochondrial fission, autophagy activation and BMPR2/Id1 downregulation. A, PASMCs were transfected with Drp1 siRNA or NC siRNA for 24 h or 48 h to examine Drp1 mRNA level by qRT‐PCR and protein level by immunoblotting, respectively. B, MitoTracker Red fluorescence staining (upper panel) of PASMCs pre‐treated with 10 μM U0126 for 30 min, or transfected with Drp1 siRNA or NC siRNA for 24 h, and then incubated with 30 ng/mL HMGB1 for 24 h, scale bar = 10 μm. Mitochondrial structure (lower panel) of PASMCs was observed by transmission electron microscopy (TEM), scale bar = 100 nm. M, mitochondria. C, PASMCs were transfected with Drp1 siRNA or NC siRNA for 24 h, and then stimulated with 30 ng/mL HMGB1 for 24 h, Beclin1, LC3B, p62, BMPR2 and Id1 protein levels were examined by immunoblotting. D, Overview of the ultrastructure of autophagosome by TEM, scale bar = 100 nm. AV, autophagic vacuoles. *P < .05

We next explored the specific changes of mitochondrial dynamin‐related protein Drp1 upon HMGB1 simulation in PASMCs. As shown in Figure 1D, HMGB1 (10‐300 ng/mL) dose‐dependently increased Drp1^Ser616^ phosphorylation in PASMCs at 1 h, and no statistical significance was found in cells treated with low concentration of HMGB1 (3 ng/mL). Figure 1E showed that HMGB1 time‐dependently increased p‐Drp1 level with the maximal effect at 1 h. However, total Drp1 expression remained unchanged (Figure 1F). Moreover, mitochondria morphology was dramatically altered, characterized by the predominance of small spherical elements in HMGB1 group compared with typical rod‐shaped and elongated mitochondria in control group detected by MitoTracker fluorescence staining and TEM (Figure 2B). Altogether, these results indicate that HMGB1 increases Drp1^Ser616^ phosphorylation and mitochondrial fragmentation.

### 3.2 ERK1/2 mediates HMGB1‐induced Drp1 phosphorylation, autophagy activation, BMPR2/Id1 downregulation and PASMCs proliferation/migration

It has been reported that activation of ERK1/2 mediates Drp1 phosphorylation and fragmented mitochondrial network, which further promotes cancer cells proliferation and chemoresistance.^17^, ^18^, ^34^ As shown in Figure 1G, incubation of PASMCs with HMGB1 caused a remarkable increase in phosphorylation level of ERK1/2. Pre‐treatment of cells with ERK1/2 inhibitor U0126 notably mitigated HMGB1‐induced Drp1 phosphorylation and restored mitochondria morphology with elongations of mitochondria (Figure 2B), indicating that ERK1/2 activation mediates HMGB1‐induced Drp1 phosphorylation and mitochondrial fragmentation in PASMCs.

We next investigated whether HMGB1 facilities autophagy activation in PASMCs. As shown in Figure 1H, HMGB1 increased Beclin1 and LC3B expressions, and decreased p62 expression. Furthermore, the number of autophagosomes was increased in HMGB1‐stimulated cells compared with control group detected by TEM (Figure 2D). These results suggest that HMGB1 promotes autophagy activation. In addition, pre‐treatment with ERK1/2 inhibitor U0126 significantly reduced HMGB1‐induced the elevations of Beclin1 and LC3B expressions, and reversed the decreased p62 expression caused by HMGB1 (Figure 1H), and reduced the number of autophagosomes (Figure 2D), implying that ERK1/2 activation mediates HMGB1‐induced autophagy activation.

**FIGURE 3:**
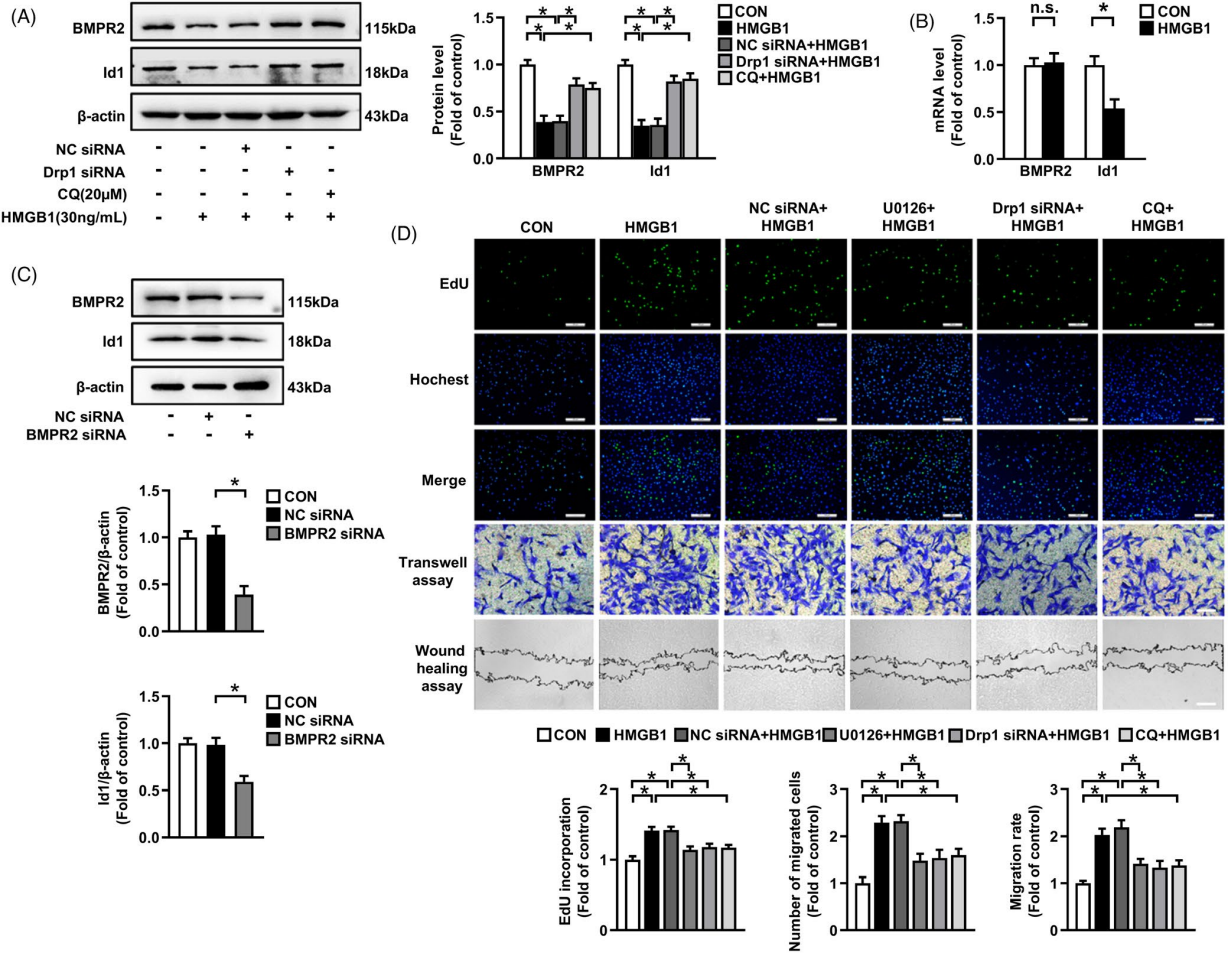
HMGB1‐induced Drp1 activation reduces BMPR2 and Id1 expressions by triggering autophagy activation and ERK1/2/Drp1/Autophagy/BMPR2/Id1 axis mediates HMGB1‐induced PASMCs proliferation and migration. A, PASMCs were transfected with Drp1 siRNA or NC siRNA for 24 h, or pre‐treated with 20 μM CQ for 1 h, and then stimulated with 30 ng/mL HMGB1 for 24 h. Protein levels were examined by immunoblotting. B, PASMCs were treated with 30 ng/mL HMGB1 for 24 h, and mRNA levels of BMPR2 and Id1 were determined by qRT‐PCR. C, Cells were transfected with BMPR2 siRNA or NC siRNA for 48 h. BMPR2 and Id1 protein levels were measured by immunoblotting. D, PASMCs were transfected with Drp1 siRNA or NC siRNA for 24 h, or pre‐treated with 10 μM U0126 for 30 min or 20 μM CQ for 1 h, and then stimulated with 30 ng/mL HMGB1 for 24 h. Cell proliferation was evaluated using EdU incorporation assay (scale bar = 200 μm), cell migration were determined by transwell assay (scale bar = 200 μm) and wound healing assay (scale bar = 400 μm). Quantitative analysis of EdU positive cells, Transwell assay and Wound healing assay, respectively. *P < .05

BMPR2/Id signalling has been indicated to be involved in the development of PAH.^22^ As shown in Figure 1H, the downregulation of BMPR2 and Id1 expressions caused by HMGB1 were reversed by ERK1/2 inhibitor U0126 pre‐treatment (Figure 1H), demonstrating that HMGB1 reduces BMPR2 and Id1 expressions through ERK1/2 activation. In addition, inhibition of ERK1/2 by U0126 significantly suppressed HMGB1‐induced PASMCs proliferation and migration (Figure 3D). Our results suggest that ERK1/2 plays an important mediator in HMGB1‐induced Drp1 activation and Drp1‐dependent mitochondrial fragmentation, autophagy activation, downregulation of BMPR2 and Id1, and PASMCs proliferation/migration.

Since extracellular HMGB1 binds to several receptors, including receptor for advanced glycation end products (RAGE) and Toll‐like receptor 4 (TLR4), to promote inflammation and tumour growth, we explored whether RAGE or TLR4 mediated HMGB1‐induced ERK1/2 and Drp1 activation and PASMCs proliferation/migration. Pre‐treatment of cells with TLR4 inhibitor TAK‐242 effectively reduced HMGB1‐induced phosphorylation of ERK1/2 and Drp1, while pre‐treatment with RAGE inhibitor FPS‐ZM1 did not significantly affect these changes caused by HMGB1 (Figure S1A). Additionally, inhibition of TLR4 attenuated HMGB1‐stimulated proliferation and migration of PASMCs (Figure S1B). Collectively, these results indicate that TLR4 majorly mediates HMGB1‐induced ERK1/2/Drp1 activation and PASMCs proliferation and migration.

### 3.3 Drp1 mediates HMGB1‐induced autophagy activation, BMPR2/Id1 downregulation and PASMCs proliferation/migration

To investigate whether hyper‐activated Drp1 was involved in HMGB1‐induced autophagy activation in PASMCs, Drp1 was silenced with sequence specific siRNA. Figure 2A showed there was a prominent reduction in Drp1 expression after knockdown, and loss of Drp1 effectively lengthened the mitochondrial morphology (Figure 2B).

Next, we investigated the downstream effectors of HMGB1‐induced Drp1‐dependent mitochondrial fission conferring to PASMCs behaviours. Based on the studies that Drp1‐dependent aberrant mitochondrial fission markedly promotes cancer cells proliferation by increasing macro‐autophagy,^17^, ^35^ siRNA was used to silence Drp1 and expressions of autophagy regulatory proteins and formation of autophagosomes were detected. We found that loss of Drp1 suppressed HMGB1‐induced Beclin1 and LC3B upregulations, p62 downregulation (Figure 2C), and the number of autophagosomes (Figure 2D), suggesting that Drp1 mediates HMGB1‐triggered autophagy activation. In addition, the decreased BMPR2 and Id1 expressions caused by HMGB1 were also preserved when PASMCs were pre‐treated with Drp1 siRNA (Figure 2C).

We further indicated that Drp1 siRNA transfection significantly suppressed PASMCs proliferation and migration caused by HMGB1 (Figure 3D), suggesting that Drp1 specifically mediates HMGB1 induction of PASMCs proliferation and migration.

### 3.4 Drp1 mediates HMGB1 induction of BMPR2 and Id1 downregulation by autophagy activation

To examine whether Drp1‐driven autophagy activation mediates HMGB1‐induced BMPR2 and Id1 defects in PASMCs, we pre‐silenced Drp1 or applied lysosomal inhibitor CQ. As shown in Figure 3A, pre‐treatment of cells with Drp1 siRNA or CQ abolished HMGB1‐induced BMPR2 and Id1 reductions. We further noticed that HMGB1 did not impact BMPR2 mRNA level but reduced Id1 mRNA level (Figure 3B). To verify whether Id1 was a direct downstream target of BMPR2 in PASMCs, BMPR2 was silenced with specific siRNA. We found that loss of BMPR2 down‐regulated Id1 expression (Figure 3C). Taken together, these results indicate that HMGB1‐induced autophagy activation results in an obvious BMPR2 lysosomal degradation and subsequent Id1 downregulation. In addition, we found that knockdown of Drp1 or inhibition of autophagy significantly suppressed HMGB1‐triggered PASMCs proliferation and migration (Figure 3D). Overall, these results suggest that Drp1‐mediated mitochondrial fission notably activates HMGB1‐induced autophagy, which further leads to BMPR2 lysosomal degradation and Id1 downregulation, thus to promote PASMCs proliferation and migration.

### 3.5 HMGB1 inhibitor GLY attenuates vascular remodelling in MCT‐induced PAH model by suppressing ERK1/2/Drp1/autophagy/BMPR2/Id1 axis

**FIGURE 4:**
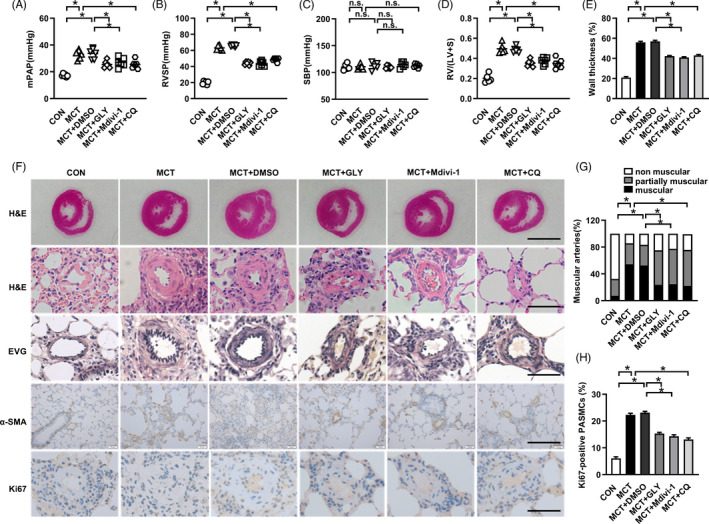
Inhibition of HMGB1 or Drp1 or autophagy alleviates the development of MCT‐induced rats PAH model. A, Changes of mPAP. B, Changes of RVSP. C, Changes of SBP. D, Changes of Fulton index (RV/(LV+S)). E, Wall thickness quantification. F, RV hypertrophy shown by haematoxylin and eosin (HE) staining, scale bar = 5 mm; Wall thickness revealed by HE staining and Eastic Van Gieson (EVG) staining, scale bar = 50 µm; Muscularization shown by α‐smooth muscle actin (α‐SMA) staining, scale bar = 200 µm; Ki67 staining showed cell proliferation, scale bar = 50 µm. G, Muscularization quantification. H, Quantitative analysis of Ki67‐positive cells. mPAP: mean pulmonary arterial pressure; RVSP: right ventricle systolic pressure; SBP: systemic blood pressure. *P < .05

Based on the above cell experiments, we generated MCT‐induced PAH rats model to further verify whether the similar mechanisms were also implicated in the development of PAH. HMGB1 inhibitor GLY reduced the increases in mPAP and RVSP in rats PAH model (Figure 4A‐B). Moreover, the elevations in RV/LV+S, pulmonary arterioles wall thickness, muscularized arteries, and PASMCs proliferation in PAs were suppressed in GLY‐treated MCT‐PAH rats (Figure 4D‐H). The levels of SBP did not change between groups (Figure 4C). Collectively, HMGB1 inhibitor GLY treatment prevented vascular remodelling and development of PAH rats model.

**FIGURE 5:**
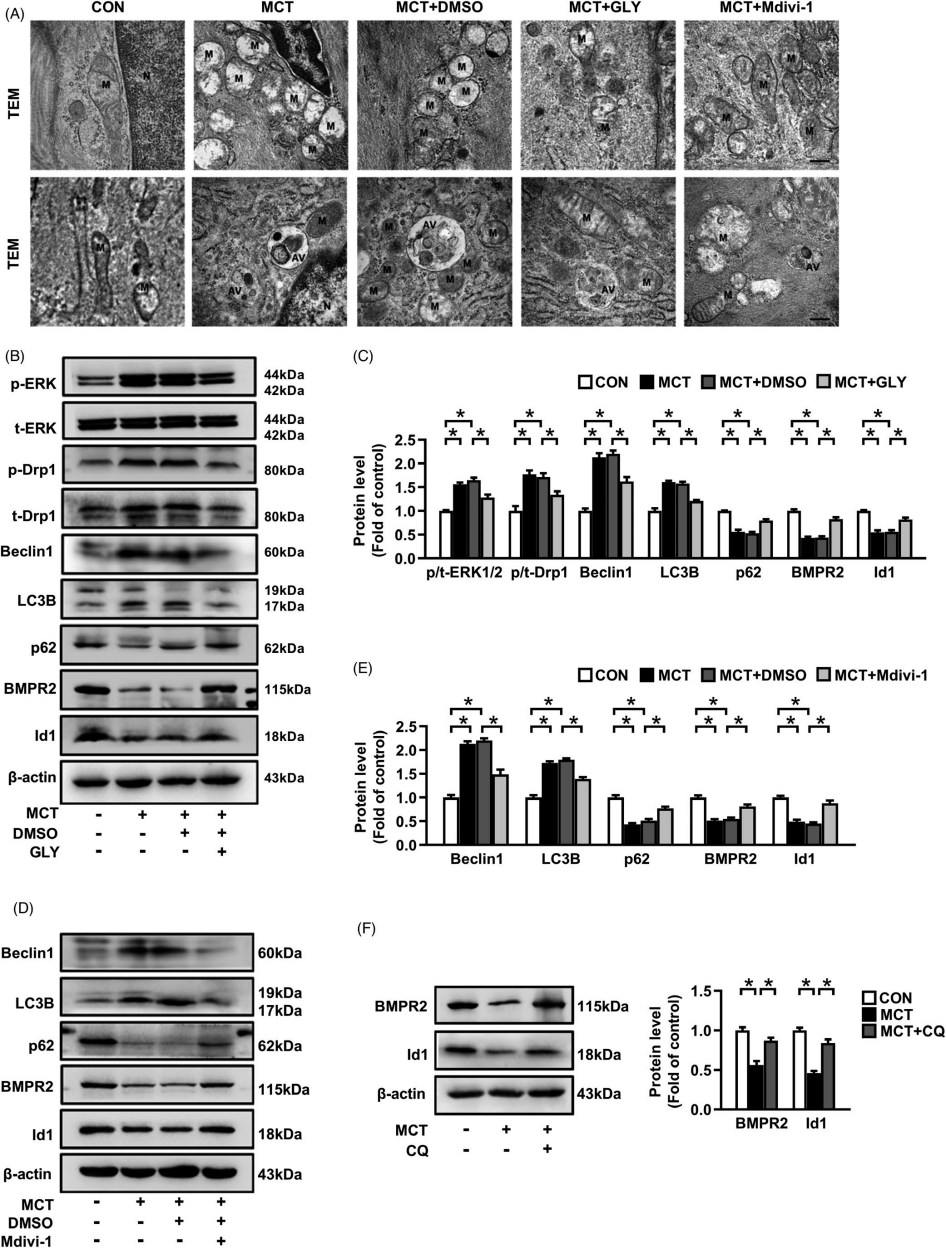
Targeting HMGB1 or Drp1 or autophagy prevents their downstream molecules expressions in MCT‐induced PAH rats. A, Overview of the ultrastructure of mitochondria (upper panel) and autophagosome (lower panel) by transmission electron microscopy (TEM), scale bar = 100 nm. M, mitochondria. N, nuclear. AV, autophagic vacuole. B, C, Proteins levels of p‐Drp1, t‐Drp1, Beclin1, LC3B, p62, BMPR2 and Id1 in lung tissues were assessed by immunoblotting. D, E, Protein levels of Beclin1, LC3B, p62, BMPR2 and Id1 in lung tissues. F, Protein levels of BMPR2 and Id1 in lung tissues. *P < .05

Next, we examined the phosphorylation levels of ERK1/2 and Drp1 in lungs of MCT‐PAH rats, and the results demonstrated that the phosphorylation of ERK1/2 and Drp1^Ser616^ were highly increased (Figure 5B,C). In addition, as shown in Figure 5A, MCT induced mitochondrial swelling, disordered or absent mitochondrial cristae structure, shorter mitochondrial length in PASMCs compared with control group. Meanwhile, autophagy activation was observed in PAH rats with increased Beclin1 and LC3B expressions, and decreased p62 expression, and increased numbers of autophagosomes (Figure 5A‐C). The BMPR2 and Id1 expressions were also reduced in MCT‐PAH rats (Figure 5B,C). However, after GLY administration, the above changes were reversed in PAH rats (Figure 5A‐C).

### 3.6 Inhibition of mitochondrial fission and autophagy suppresses pulmonary vascular remodelling and the development rats PAH by restoring BMPR2 and Id1 expressions

To determine whether Drp1‐dependent mitochondrial fission and consequent autophagy activation mediate pulmonary vascular remodelling and the development of PAH, Mdivi‐1, a widely used small molecule to inhibit mitochondrial fission, and autophagy blocker CQ were applied in MCT‐induced PAH rats. Compared to MCT group, administrations of Mdivi‐1 or CQ effectively reduced mPAP and RVSP, while had no significant effect on SBP (Figure 4A‐C), respectively. Additionally, the increases in right ventricular hypertrophy, pulmonary arterioles wall thickness, muscularized arteries and PASMCs proliferation were alleviated in Mdivi‐1‐treated and CQ‐treated PAH rats (Figure 4D‐H).

We next detected the changes of mitochondrial structure by TEM and autophagy activity in PAH rats with the treatment of Mdivi‐1. Results showed that Mdivi‐1 treatment inhibited MCT‐induced mitochondrial alterations (Figure 5A) and blocked autophagy activation indicated by upregulation of p62, downregulation of Beclin1 and LC3B, and the decreased number of autophagosomes (Figure 5A,D,E). In addition, Mdivi‐1 also increased BMPR2 and Id1 expressions in MCT‐PAH model (Figure 5D,E). Similarly, administration of CQ also reversed BMPR2 and Id1 downregulation (Figure 5F). Together, these findings indicate that inhibition of Drp1 suppresses autophagy activation, which further blocks BMPR2 lysosomal degradation and Id1 downregulation, thus to prevent MCT‐induced PAH development.

## 4 DISCUSSION

In the present study, we consolidated the causal role of HMGB1 in PAH and elucidated the mechanism underlying HMGB1‐induced pulmonary vascular remodelling in PAH. We demonstrated that HMGB1 increased Drp1 phosphorylation and Drp1‐dependent mitochondrial fission through activation of ERK1/2 signalling pathway, and subsequently stimulated autophagy activation, which further led to BMPR2 lysosomal degradation and Id1 downregulation, and ultimately promoted PASMCs proliferation/migration and pulmonary vascular remodelling in PAH.

Extracellular HMGB1 is released by damaged cells and functions as a damage‐associated molecular pattern that regulates various cellular processes, including cell proliferation, migration and differentiation.^17^, ^36^ Circulating HMGB1 levels are increased in PAH patients and PAH animal models, and these elevations correlate with disease severity.^4^, ^6^ Our study confirmed that HMGB1 promoted proliferation and migration of PASMCs, and inhibition of HMGB1 by GLY prevented pulmonary vascular remodelling in MCT‐induced PAH model, which are consistent with previous studies.^8^, ^9^, ^10^, ^11^

Drp1, a member of the dynamin family of GTPases, is a pivotal component for mitochondrial division. When activated, Drp1 translocates into mitochondria and triggers mitochondrial fission. Hyper‐activated Drp1 and excessive mitochondrial fission, which results in mitochondrial fragmentation, is a new hallmark of proliferative diseases, including cancers and PAH.^37^, ^38^, ^39^ Activation of Drp1 and induction of Drp1‐dependent mitochondrial fission have been reported in PASMCs from PAH patients and shown to participate in PAH development by promoting PASMCs proliferation.^12^, ^14^ Our study showed that HMGB1 significantly increased Drp1 phosphorylation in PASMCs accompanied with elevated fragmented mitochondria. We further indicated that knockdown of Drp1 inhibited HMGB1‐induced proliferation and migration of PASMCs, this is consistent with the results reported in other cell types.^17^, ^40^ In addition, HMGB1 inhibitor GLY administration prevented PAH development by suppressing Drp1 activation and Drp1‐dependent mitochondrial fission in MCT‐PAH model. Our results indicate that Drp1 mediates HMGB1‐induced PASMCs proliferation/migration and pulmonary arterial remodelling.

We found that the protein level of Drp1 was not changed in HMGB1‐treated PASMCs, indicating that HMGB1‐induced Drp1 activation is caused by posttranslational modification of Drp1. Phosphorylation is one of the most critical posttranslational modifications which alters Drp1 activity. Drp1 is phosphorylated by several kinases, including ERK1/2, which activates Drp1 by phosphorylation of serine 616, leading to aberrant mitochondrial fission.^18^, ^41^, ^42^ The ERK1/2 pathway plays a key role in controlling cell proliferation, migration, differentiation and survival.^43^ Activation of ERK1/2 has been found in patients with PAH and PAH animal models.^44^, ^45^ In this study, we observed that HMGB1 significantly increased the phosphorylation of ERK1/2, which further mediated HMGB1‐induced Drp1 activation and Drp1‐dependent mitochondrial fissions, thus to promote PASMCs proliferation/migration and pulmonary arterial remodelling. These results are in agreement with previous reports demonstrated in cancer cells.^17^, ^46^ Moreover, it has been shown that TLR4 and RAGE receptors are implicated in HMGB1‐induced vascular remodelling,^6^, ^47^, ^48^, ^49^ and we found that TLR4 was the predominant receptor that mediated HMGB1‐induced ERK1/2 and Drp1 activation. Taken together, our study suggests that ERK/Drp1 pathway plays an important role in HMGB1‐induced PASMCs proliferation/migration and pulmonary vascular remodelling.

We showed that HMGB1 increased Drp1^Ser616^ phosphorylation, but did not change Drp1 expression in cultured PASMCs. However, both phosphorylation of Drp1 at Ser616 and expression of Drp1 were elevated in MCT‐PAH rats. The difference of Drp1 expression between in vitro and in vivo might due to several reasons. Firstly, HMGB1‐induced Drp1 activation is caused by posttranslational modification of Drp1 in PASMCs. Secondly, a variety of bioactive mediators are over‐produced and multiple pro‐proliferative/anti‐apoptotic signalling cascades are activated in MCT‐induced rat PAH model. These might lead to the discrepancy of t‐Drp1 expression between in vitro and vivo. We also observed that t‐Drp1 was upregulated in MCT‐PAH rats and decreased after GLY treatment. GLY is a multi‐targeted compounds and HMGB1 is one of the main targets of GLY.^50^ Previous studies have reported that GLY interacts with the Nrf2‐binding site of Keap1 and competitively inhibits Keap1‐Nrf2 interaction, which subsequently activates Nrf2 pathway,^51^, ^52^, ^53^ thus to promote the degradation of Drp1.^54^, ^55^ This might result in the decreased t‐Drp1 expression in PAH rats after GLY treatment. Further studies with more specific HMGB1 inhibitors or HMGB1 knockout animal model will be important for providing additional evidence to support the data presented here.

Autophagy is a highly regulated catabolic process that involves sequestration and lysosomal degradation of cytosolic components including dysfunctional organelles and misfolded proteins, which is activated by stress conditions including hypoxia, reactive oxygen species, inflammation and DNA damage.^20^ Extracellular HMGB1 has been found to be a potent inducer of autophagy and promote cell survival, migration, and chemoresistance.^56^, ^57^ In the present study, we showed that HMGB1‐triggered autophagy activation through ERK1/2‐mediated Drp1 phosphorylation in PASMCs. Our in vivo study further confirmed that inhibition of Drp1 by Mdivi‐1 alleviated pulmonary vascular remodelling in MCT‐PAH model by inhibiting autophagy activation and autophagosomes formation. Moreover, recent studies have demonstrated that Drp1 activation and subsequent mitochondrial fission significantly promoted cancer cells proliferation and chemoresistance by increasing autophagy.^17^, ^18^, ^28^ Our study indicates that ERK/Drp1‐mediated mitochondrial fission is responsible for HMGB1‐induced autophagy activation, resulting in PASMCs proliferation and migration.

BMPR2 is a transmembrane serine/threonine kinase receptor of the bone morphogenetic protein (BMP) that mediates the activation of intracellular Smad downstream effectors and subsequently down‐regulates Id1, which further controls cell proliferation and differentiation.^21^ Apart from heterozygous mutations in BMPR2 gene in familial PAH and idiopathic PAH, the reductions in BMPR2 levels and activity have also been found to be involved in non‐genetic forms of PAH.^58^ Recently, it has been reported that endogenous BMPR2 is degraded through the TNF‐α‐induced autophagy activation and inhibition of lysosomal degradation results in BMPR2 accumulation at plasma membrane in PAECs.^20^ Our study demonstrated that HMGB1 reduced BMPR2 through Drp1‐mediated autophagy activation, and loss of BMPR2 further down‐regulated Id1 expression and contributed to PASMCs proliferation and migration. Pharmacological inhibition of autophagy restored the decreased BMPR2 and Id1 protein levels in MCT‐induced rats PAH model, which is consistent with previous studies.^19^, ^20^, ^22^ Taken together, our study suggest that HMGB1 induces autophagy by ERK1/2‐mediated Drp1 activation and subsequent mitochondrial fission, which further reduces BMPR2/Id1 expressions and therefore promotes proliferation and migration of PASMCs and consequently pulmonary vascular remodelling.

The major limitations of the present study are that only rat PASMCs and MCT‐induced PAH rats model are used to explore the role of HMGB1 induction of PASMCs proliferation/migration and pulmonary arterial remodelling. Primary human PASMCs (hPASMCs) or PASMCs from PAH patients are considered to be the most suitable cells to investigate the pathophysiology of pulmonary arteries under normal and disease conditions. However, it has been proven to be inconvenient, expensive and difficult to obtain PASMCs from humans. Rats have been widely used for PAH study in vivo and in vitro, which provide important information for understanding the mechanisms of development of PAH. In addition, there are numerous animal models of PAH and no single preclinical model can completely recapitulate the diverse forms of PAH.^59^ Due to reproducibility, low cost and simple technical skills, MCT model has been successfully applied in the elucidation of the molecular mechanisms related to PAH.^59^, ^60^ MCT‐induced PAH model exhibits the initial phase of PAH pathogenesis, including medial and adventitial thickening of the pulmonary artery, muscularization of small and normally non‐muscularized small pulmonary arteries, and an aberrant inflammatory cell response.^59^, ^61^ Further studies by using cultured primary hPASMCs or PASMCs from PAH patients, and multiple animal models or transgenic animals would rigorously allow us to corroborate the role of HMGB1/Drp1/autophagy axis in vascular remodelling progress of PAH.

## 5 CONCLUSIONS

In this study, we highlight the crucial role of HMGB1 in PAH and have demonstrated that HMGB1 promotes PASMCs proliferation/migration and pulmonary vascular remodelling by activating ERK1/2/Drp1/Autophagy/BMPR2/Id1 axis. Targeting HMGB1 signalling pathway might have potential value in therapeutic intervention of PAH.

## CONFLICT OF INTEREST

The authors declare that they have no conflict of interest.

## AUTHOR CONTRIBUTIONS

The conception and design were proposed by WF and ML. Cell and animal experiments were finished by WF, JW, XY and LC. Molecular biology experiments were performed by WF and QZ. Data analysis were conducted by WF, QW and WS. Data collection were carried out by WF, YC, JL and ZQ. Paper was drafted by WF and reviewed by SL, XM and ML. All authors read and approved the manuscript.

## Supporting information

Fig S1

Supplementary Material

## Data Availability

Data are available from corresponding author upon reasonable request.
