# Supplementary figures and images for: ERK/Drp1‐dependent mitochondrial fission contributes to HMGB1‐induced autophagy in pulmonary arterial hypertension
Source: Cell Prolif. 2021 May 4;54(6):e13048. doi: 10.1111/cpr.13048 (PMC8168414; doi:10.1111/cpr.13048)

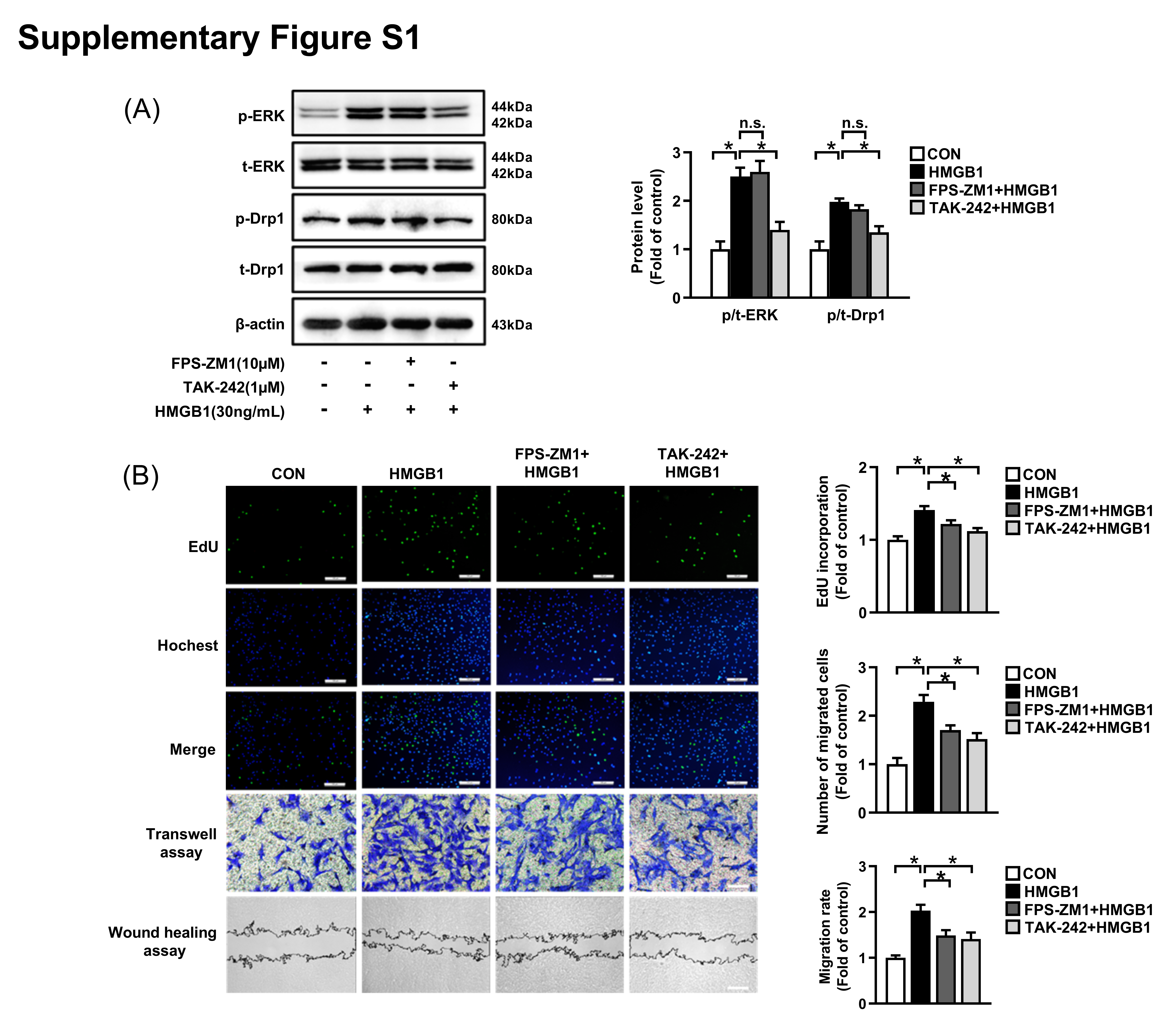

Supplement: Supplementary file 1 — Fig S1 [file CPR-54-e13048-s001.tif]
