# Supplementary material for: ERK/Drp1‐dependent mitochondrial fission contributes to HMGB1‐induced autophagy in pulmonary arterial hypertension
Source: Cell Prolif. 2021 May 4;54(6):e13048. doi: 10.1111/cpr.13048 (PMC8168414; doi:10.1111/cpr.13048)
Supplement: Supplementary file 2 — Supplementary Material [file CPR-54-e13048-s002.docx]

**Figure legends**

**Supplementary Figure 1.** TLR4 mediates HMGB1-induced ERK1/2 and Drp1 activation, PASMCs proliferation and migration. A, PASMCs were pre-treated with 10 μM FPS-ZM1 or 1 μM TAK-242 for 30 min, and then stimulated by 30 ng/mL HMGB1, the phosphorylation levels of ERK1/2 and Drp1, and total ERK1/2 and Drp1 were examined by immunoblotting. B, Cell proliferation was evaluated using EdU incorporation assay (scale bar= 200 μm), cell migration were determined by transwell assay (scale bar= 200 μm) and wound healing assay (scale bar= 400 μm). **P* < 0.05.
